# Epigenetics: the panacea for cognitive decline?

**DOI:** 10.18632/aging.101366

**Published:** 2018-01-18

**Authors:** Mahima Sharma, Radha Raghuraman, Sreedharan Sajikumar

**Affiliations:** 1 Department of Physiology, National University of Singapore, Singapore; 2 Neurobiology/Aging Programme, Life Sciences Institute, Centre for Life Sciences, Singapore

**Keywords:** epigenetics, G9a/GLP complex, Alzheimer’s disease, aging, memory

Age-related neurodegenerative decline is on the rise, Alzheimer’s disease (AD) being the most prevalent form of dementia amongst the aging population worldwide. The current number of 46.8 million AD-affected people is expected to reach 131.5 million by 2050. The AD and the associated cognitive decline places a huge socio-economic burden on the society at large. Though the hunt for therapeutic interventions to tackle the cognitive decline has been on the pursuit for 30 years, we are yet to see the light at the other end of the tunnel.

Epigenetics (changes in the gene expression or functionality without any change in the DNA sequence) confers a promising approach to improve the cognitive performance in the AD individuals. The most common forms of epigenetic changes are-DNA methylation and post-translational modification of histone tails-whether it be acetylation, methylation, phosphorylation and the like. Histone deacetylase (HDAC) inhibitors are emerging as potential pharmacological agents to treat AD [1]. The results from our study add another feather to the cap. Our study identifies G9a/G9a like protein (GLP) histone dimethyltransferase complex as another epigenetic factor that underlies the development of Alzheimer’s disease [2]. Inhibition of the enzymatic disruption of this complex improves the deficits in long-term potentiation (LTP- a cellular correlate of memory) and the associative mechanisms (studied by Synaptic tagging and Capture (STC)) in AD model. Associative memory is severely impaired during mild cognitive impairment (MCI), which plays harbinger of AD [2]. Improvement of associative memory on a synaptic scale by the modulation of G9a/GLP complex makes it a well suited target in AD interventions.

Brain derived neurotrophic factor (BDNF), one of the plasticity proteins, is crucial for synaptic plasticity, which forms the basis of learning and memory [3]. Its lower levels are associated with poor cognition [4]. Inhibition of G9a/GLP activity releases the brake on the transcription of BDNF and thereby increases the BDNF level in the neuron that then maintains different forms of synaptic plasticity and associativity. These results open up realms for the epigenetic research to delve further into the G9a/GLP complex dynamics during the progression of AD [2]. Does modulation of G9a/GLP activity improve the cognitive performance of different AD mice models? If yes, does the cognitive improvement in these mice relate to the upregulation of BDNF or to any other plasticity proteins? The answers to these questions will bear testimony to the candidature of G9a/GLP inhibitors as the AD-tackling agents.

Moreover, epigenetic marks are dynamically regulated and changes are exerted in their profile depending on various environmental factors- be it stress, drug-addiction, exercise, nutrition or environmental enrichment. Stress and drug addiction are known to change the levels of G9a/GLP complex [5]. There has been an inclination towards the usage of non-pharmacological methods like exercise [6] and mindfulness [7] to ameliorate the cognitive inefficiency in AD patients. It is pivotal to observe the profile of various epigenetic marks in the event of environmental factors including exercise, mindfulness, environmental enrichment and diet. Given the extensive time and effort it takes for the pharmacological interventions to address even a fraction of these complications, non-invasive method of addressing aging related cognitive deficits has a farther reach with regards to encompassing candidates of a broader socioeconomic status. In this regard, it is pivotal that the epigenetic hypothesis of aging related cognitive deficits needs to be embraced with greater significance as epigenetics has crawled its way into the human behaviour and cognition as the fundamental regulator of learning and memory.
